# Nicotinamide mononucleotides alleviated neurological impairment via anti-neuroinflammation in traumatic brain injury

**DOI:** 10.7150/ijms.80942

**Published:** 2023-01-31

**Authors:** Xiaolu Zhu, Jin Cheng, Jiangtao Yu, Ruining Liu, Haoli Ma, Yan Zhao

**Affiliations:** 1 Emergency Center, Zhongnan Hospital of Wuhan University, Wuhan, China.; 2 Department of Biological Repositories, Zhongnan Hospital of Wuhan University, Wuhan, China.; 3 Hubei Clinical Research Center for Emergency and Resuscitation, Zhongnan Hospital of Wuhan University, Wuhan, China.

**Keywords:** Traumatic brain injury, Nicotinamide mononucleotides, anti-neuroinflammation, neuronal injury, NF-kappa B

## Abstract

Traumatic brain injury (TBI) is one of the main factors of death and disability in adults with a high incidence worldwide. Nervous system injury, as the most common and serious secondary injury after TBI, determines the prognosis of TBI patients. NAD^+^ has been confirmed to have neuroprotective effects in neurodegenerative diseases, but its role in TBI remains to be explored. In our study, nicotinamide mononucleotides (NMN), a direct precursor of NAD^+^, was used to explore the specific role of NAD^+^ in rats with TBI. Our results showed that NMN administration markedly attenuated histological damages, neuronal death, brain edema, and improved neurological and cognitive deficits in TBI rats. Moreover, NMN treatment significantly suppressed activated astrocytes and microglia after TBI, and further inhibited the expressions of inflammatory factor. Besides, RNA sequencing was used to access the differently expressed genes (DEGs) and their enriched (Kyoto Encyclopedia of Genes and Genomes) KEGG pathways between Sham, TBI, and TBI+NMN. We found that 1589 genes were significantly changed in TBI and 792 genes were reversed by NMN administration. For example, inflammatory factor CCL2, toll like receptors TLR2 and TLR4, proinflammatory cytokines IL-6, IL-11 and IL1rn which were activated after TBI and were decreased by NMN treatment. GO analysis also demonstrated that inflammatory response was the most significant biological process reversed by NMN treatment. Moreover, the reversed DEGs were typically enriched in NF-Kappa B signaling pathway, Jak-STAT signaling pathway and TNF signaling pathway. Taken together, our data showed that NMN alleviated neurological impairment via anti-neuroinflammation in traumatic brain injury and the mechanisms may involve TLR2/4-NF-κB signaling.

## Introduction

Traumatic brain injury (TBI), as the most common cause of death in trauma centers, is also one of the major causes of death and disability in adults worldwide 1, 2. Globally, the annual incidence rate of TBI is about 50 million 3, and more than 700,000 TBI incidents have been reported every year in China, of which severe TBI led to more than a quarter of the mortality rate and half of the adverse consequences 4. Car crashes, falls, acceleration/deceleration and assaults are common causes of TBI. Therefore, the only possible treatment of primary injury is prevention, such as reducing dependence on motor vehicles and wearing helmets properly 5, 6. Secondary injury in the seconds, minutes, hours, days is caused by a complex series of pathophysiological reactions, such as excitotoxicity, mitochondrial dysfunction, apoptosis, autophagy and inflammation, which will cause irreversible damage to neurons 7, 8. There are several follow-up managements for TBI. At present, surgery such as debridement and inhibition of intracranial pressure target for penetrating TBI of acute stage 3; drugs like diuretics and anticonvulsants used for preventing hydrocephalus and seizures in modest TBI 9; physical therapy can relieve muscle spasms and contractions in mild TBI 10, but their effects on secondary injuries are limited 11. Previous study has shown that Aucubin can reduce the inflammatory in mice after TBI to exert neuroprotective effect 12. Omega-3 can reduce the loss of neurons by reducing the expression of inflammatory and apoptotic factors 13.

NAD^+^, as a key coenzyme in eukaryotic organisms, determines hundreds of enzymatic reactions 14. In the most basic energy metabolism process, reduced and oxidized NAD are indispensable factors as hydrogen ion transfer carriers 15. In addition, NAD^+^ can also indirectly regulate the process of anti-stress, cell growth, anti-inflammatory and anti-aging processes by regulating the function of Sirtuins factor which has NAD^+^ dependent protein deacetylation activity 16. Previous studies have shown that the decline of NAD^+^ level will also lead to heart failure and Alzheimer's disease 17. *In vivo* models of excitotoxic injury, the death of neurons is usually accompanied by a decrease in NAD^+^ levels 18. In addition, the study showed that moderate and severe TBI could lead to a decrease in the level of NAD^+^ in the brain 19.

Nicotinamide mononucleotides (NMN) is the direct precursor of NAD^+^. It has been used in many medical researches because it can rapidly and effectively increase the level of NAD^+^ 14. NMN is currently being used in the research of various neurological diseases, and has been observed to have neuroprotective properties and improve neurological function 20-24. In cerebral ischemia mice, NMN showed neuroprotective effects though improving mitochondrial metabolism and alleviating oxidative stress injury 24. Besides, NMN can also reduce the loss of neurons and inhibit the decline of cognitive function in AD mice 25.

Therefore, we propose the hypothesis that NMN may improve the behavior function as well as memory ability and alleviate the neurological damage of moderate-to-severe TBI rats. Through the treatment of Sprague-Dawley rats with TBI, the behavioral and pathological changes of rats were observed, and transcriptome sequencing of hippocampal tissues of rats was conducted to explore the role of NMN in TBI rats and the pathways which might be involved, hoping to find a new treatment for TBI.

## Methods and materials

### Animals

A total of 66 male Sprague Dawley rats, weighing 280-320g (7 weeks), were purchased from Vital River Laboratory Animal Technology (Beijing, China). The rats were housed in the Animal Experimental Center of Zhongnan Hospital at Wuhan University under 12 hours light/dark cycles with temperature controlled at 25 °C ± 2 °C. Rats had free access to standard laboratory diet and water. All animal experiments were carried out in accordance with the guidelines for experimentation with lab animals established by the Animal Experiment Center and Ethics Committee of Zhongnan Hospital of Wuhan University.

### TBI procedure

Controlled cortical impact (CCI) device (Custom Design & Fabrication, USA) was employed to establish the rat model of a moderate-to-severe traumatic brain injury as described previously 26, 27. After one week of rest, the animals were assigned randomly to one of three groups: sham group, TBI group and TBI+NMN group. Diet was stopped 12 hours before surgery. 3% Pentobarbital sodium (50mg/kg, i.p.) was injected to anesthetize rats. The scalp of rats was shaved and disinfected. The skull was exposed by incision in the middle of the skin of head. A craniotomy with a diameter of 5 mm was drilled in the right parietal region, the caudal side of the coronal and right side of the sagittal suture. The rats were placed on CCI device and their heads were fixed - the impactor was directed at the bone window and contacted with the meninges, and the parameter velocity was 5 m/s, dwell time was 200 ms and impact depth was 3 mm. The sham group received the same treatment except for impact. Analgesics and antibiotics were applied after surgery.

### The detailed experimental arrangement

**Figure 1:**
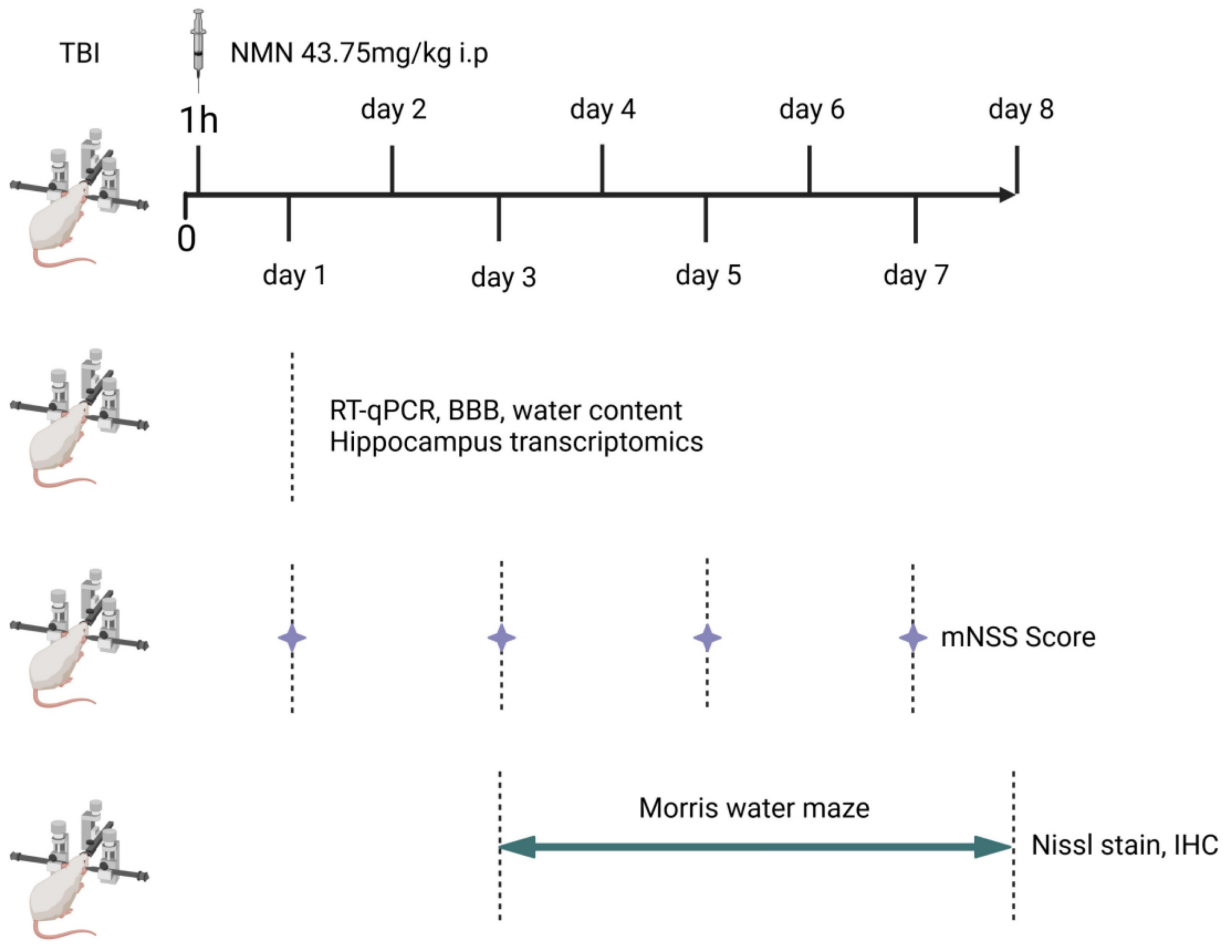
Scheme of the procedures used for experiments.

All animals were randomly divided into three groups, Sham, TBI and TBI+NMN (22 rats in each group). One hour after the operation, the TBI group was not given drugs, while the TBI+NMN group were injected with NMN and sham group were injected with vehicle (PBS). The brain tissues of some rats were collected 24 hours after TBI for sequencing, RT-qPCR, brain water content and blood brain barrier tests. The tissues were directly put into liquid nitrogen and then transferred to -80 °C for storage. The neurological deficits of the remaining rats were measured by mNSS score at 1,3,5 and 7 days after TBI. Morris water maze test was conducted from the 3^rd^ to the 8^th^ day. On the 8^th^ day, the brain was removed and stored in 4% paraformaldehyde solution (**Fig. 1**).

### NMN administration

NMN was dissolved in Phosphate Buffer Saline (PBS) with a concentration of 20 mg/ml. Rats were injected with NMN (MCE, USA., HY-F0004, 43.75mg/kg, i.p.) or vehicle (PBS, same volume, i.p.) 1 hours after TBI impact experiment 28.

### Nissl staining

After the rats were euthanized 8 days after TBI, normal saline and 4% paraformaldehyde were perfused into the left atrium to flush out the blood and fix the tissues. Three rats brain tissue in each group were obtained and fixed in paraformaldehyde. The paraffin blocks were sliced with a thickness of 10 μm. The brain was cut into three coronal sections to observe the structure of hippocampal CA1, CA3 and DG regions. After dewaxing, the slices were dyed in 0.1% cresol violet solution for 10 minutes, then washed in distilled water for three times, and dehydrated in 95% ethanol. Observe and collect images under Olympus microscope 29. Image J software was used for processing.

### mNSS Score

On the days 1, 3, 5 and 7 days after TBI, the neurological function was scored using modified neurological severity score (mNSS), and 6 rats in each group were tested each time. 30. mNSS is a comprehensive test including motor, sensory, balance and reflex (normal score, 0, maximum score, 18 points). The final points were 13-18 for severe injury, 7-12 for moderate injury, and 1-6 for mild injury. The higher the score, the more severe the nerve damage of rats. The test was conducted in double blindness and scored by three professional researchers.

### Morris water maze test

Morris water maze (MWM) test was performed on 5 rats in sham group, 6 rats in TBI and TBI+NMN groups. Animals were subjected to the Morris water maze test for the purpose of assessing spatial learning and memory 31. The rats were put into a vat of inky water with a diameter of 2.1 meters. The tank is delimited four quadrants, and a platform is placed in one quadrant, which is 1cm below the water surface. On the 3^rd^ to 7^th^ days after TBI, all rats were tested four times a day. Each time, one rat was placed in a different quadrant, and the time spent to find the platform after it was placed in water was recorded. If it exceeds 120 seconds, guide the rat to find the platform and allow it to stay on the platform for 30 seconds. Remove the platform on the sixth day, and recorded the escape latency of the rats. Each stage of the experiment was recorded by the automatic tracking system (Xmaze™, Xinruan Information Technology Co., Shanghai, China).

### Brain water content

Three rats per group were euthanized by giving 3% pentobarbital (150 mg/kg, i.p.). Brain tissues of rats were obtained after 24h post-TBI, removing the olfactory bulb and cerebellum. The brain was divided into ipsilateral (damaged) and contralateral (control) hemispheres, immediately weighed to obtain wet weight. Then drying samples for 24 hours at 100 °C in the oven to obtain the dry weight. The calculation formula of brain water content is as follows, (wet weight - dry weight)/ wet weight*100% 32.

### Immunofluorescence staining

The brains of 3 rats in each group used for immunofluorescence were taken from the rats killed after neurological function test. The brain samples were dissected in 4% paraformaldehyde (24h, 4 °C), transferred to 30% sucrose solution for dehydration (72h, 4 °C), and then frozen (- 80 °C). The frozen brain tissues were then sliced at a thickness of 10 μm coronal brain sections with cryostat microtome (Leica Microsystems, Germany). The slices were stored in a -80 °C freezer until analysis. Three sections were taken from each rat for immunofluorescence staining, and one of the sections with the best effect was shown. The brain tissue sections were placed in a repair box filled with antigen repair buffer (0.01M citric acid buffer, pH 6.0), heated to 100 °C and incubated for 15 minutes for antigen repair. Sample washed with PBS (PH 7.4) for three times, and add goat serum at RT for 30 minutes for nonspecific binding sites blocking. Then add GFAP, IBA-1, NEUN (Abcam, ab7260, ab178847, ab177487, 1:500) primary antibody and incubate them in a 4 °C wet box overnight. After PBST washing, sections were incubated with second antibody in a wet box at 37 °C for 1 hour. NEUN incubated sections, with a drip concentration of 20 μg/ml protease K solution was incubated at room temperature for 20 minutes, and then the apoptosis detection kit (Vazyme, A113-03) was used for tunel staining. DAPI (Beyotime, C1002, 1:1000) was used for the nuclear counterstaining. Fluorescence images were captured with a fluorescence microscope (Olympus, Japan) and fluorescence intensity was quantified with Image J.

### RNA-seq analysis

RNA-seq analysis was performed on 4 rats in each group. Euthanized rats were perfused 0.9% NaCl, and the brain tissues were removed. Bilateral hippocampus was separated on ice, directly put into liquid nitrogen, and transferred to - 80 °C refrigerator for preservation. Total RNA of hippocampus was extracted and purified by using Trizol reagent (Thermofisher, 15596018) based on the protocol provided by the manufacturer. RNA samples quantity and purity were analysis of Bioanalyzer 2100 and RNA 6000 Nano LabChip Kit (Agilent, CA, USA, 5067-1511), high-quality RNA samples with RIN number > 7.0 were used to construct cDNA library. Finally, we use illumina Novaseq™ 6000 (LC Bio Technology CO., Ltd. Hangzhou, China) conducts double ended sequencing (PE150) according to the supplier's recommended protocol.

To get high quality clean reads, reads were further filtered by Cutadapt, removing reads containing adapters, poly A and poly G, and unknown nucleotides bases. Then sequence quality was verified using FastQC. The reference genome was mapped using HISAT2 software. StringTie (1.3.1) was used to reconstruct transcripts and calculate the FPKM value of all gene expression levels in each sample. The differentially expressed mRNAs were used for GO enrichment and KEGG enrichment analysis, screened with | log2 (fold change) | > 0.5 and p value < 0.01. OmicStudio (https://www.omicstudio.cn/) was applied to perform the heatmap, venny analysis, GO and KEGG enrichment analysis.

### RT-qPCR confirmation

To confirm the accurate RNA sequencing, Real-Time PCR was performed on 6 rats each group. The experiments were performed on a CFX Connect Real-Time PCR System (Bio-Rad; CFX Maestro 1.0 software). The target RNA and TB Green Premix Ex Taq II (Tli RNaseH Plus) (RR820A, TaKaRa, Japan) were subjected to the system. All procedures were performed according to standard protocols. Primers used in this study were listed in Table 1.

### Statistical analysis

All data were expressed as mean ± standard deviation (SD). Difference between two groups was tested by Student's t test, and a one- or two-way analysis of variance (ANOVA) was performed for multiple groups. Values of *P* < 0.05 were considered to indicate a statistical different. Analyses were performed using the SPSS software (V25.0, IBM, United States).

## Results

### NMN treatment reduced the neurological damage and improved neurological functions after TBI

**Figure 2:**
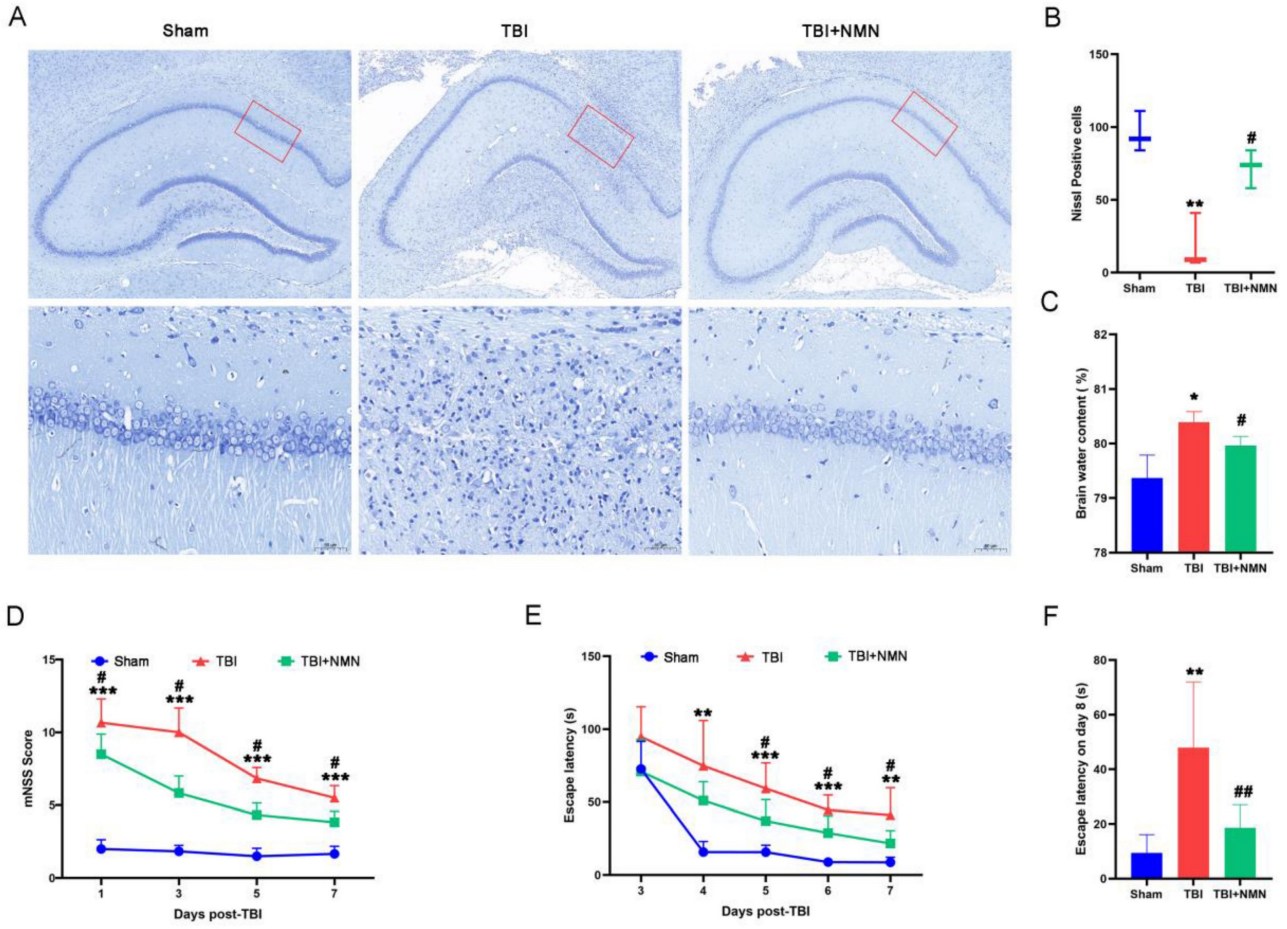
** NMN treatment reduced the neurological damage and improved neurological function after TBI. A and B)** Nissl staining at 8 days after TBI in the Brain sections (n = 3 per group). **C)** Brain water content of damaged hemisphere 1day post-TBI (n = 3 per group). **D)** mNSS score at 1, 3, 5, and 7 days following TBI/sham (n = 6 per group). **E)** Escape latency in MWM test 3 to 7 days following TBI/sham (n = 5 sham group, n=6 TBI and TBI + NMN group). **F)** Escape latency at test day following TBI/sham (*p < 0.05, **p < 0.01, and ***p < 0.001 TBI vs Sham group; #p < 0.05 and ##p < 0.01 TBI vs TBI +NMN group).

To explore the neuroprotective effect of NMN on TBI, we first examined the survival of nerve cells. As shown by Nissl staining (**Fig. 2A**), neuron structure was clear and Nissl bodies were evenly distributed in sham group, but the structure of neurons was disappeared after TBI operation. However, less loss of neurons combined with normal structure were showed in the TBI+NMN group Compared to TBI group, indicating that NMN treatment reduced the neurological damage in hippocampal CA1 area after TBI. Quantitative statistics also indicated that the numbers of Nissl-positive cells in sham group was 97 ± 14 cells/field, while the TBI group was far less than sham group (*P* < 0.01), which was 24±17 cells/field. And the numbers of Nissl-positive cells in TBI+NMN group was significantly increased compared to TBI group, which was 71 ± 13 cells/field (*P* < 0.05) (**Fig. 2B**).

In addition, the measurement of brain water content also demonstrated that the brain edema after TBI was also alleviated by NMN treatment, as the brain water content in the TBI+NMN group was significantly lower than TBI group (TBI+NMN vs TBI, 79.97% ± 0.16% vs 80.41% ± 0.19%, *P* < 0.05) (**Fig. 2C**).

In order to observe whether NMN administration affect the neurological functions after TBI, we conducted tests to measure mNSS scores and performed behavioral experiments using the Morris water maze. At 1, 3, 5, and 7 days after TBI, the mNSS scores of TBI group were significantly higher than those of sham group (all *P* < 0.001). But after NMN treatment, the mNSS scores were significantly reduced. (*P* < 0.05) (**Fig. 2E**). We also performed MWM tests at 3-8 days after surgery to investigate the effects of NMN on spatial learning and memory ability after TBI. On the first day of training, there was no evidently difference in the escape latency of the three groups. However, from 4 to 7 days after TBI, the escape latency of the TBI group was significantly higher than the sham group (*P* < 0.01 on days 4 and 7; *P* < 0.001 on days 5 and 6). Consistent with the mNSS score results, NMN treatment rats showed a significantly lower escape latency compared to TBI group during this period (*P* < 0.05) (**Fig. 2F**). Moreover, the latency in TBI+NMN group was also lower than TBI group on the test day of MWM (*P* < 0.01) (**Fig. 2G**). Taken together, these results strongly suggest that NMN has significant therapeutic effect against neurological injury and neurobehavioral damage after TBI.

### NMN reduced the numbers of reactive astrocytes and microglia, and inhibited the transcription level of TNF-α and IL-1β

**Figure 3:**
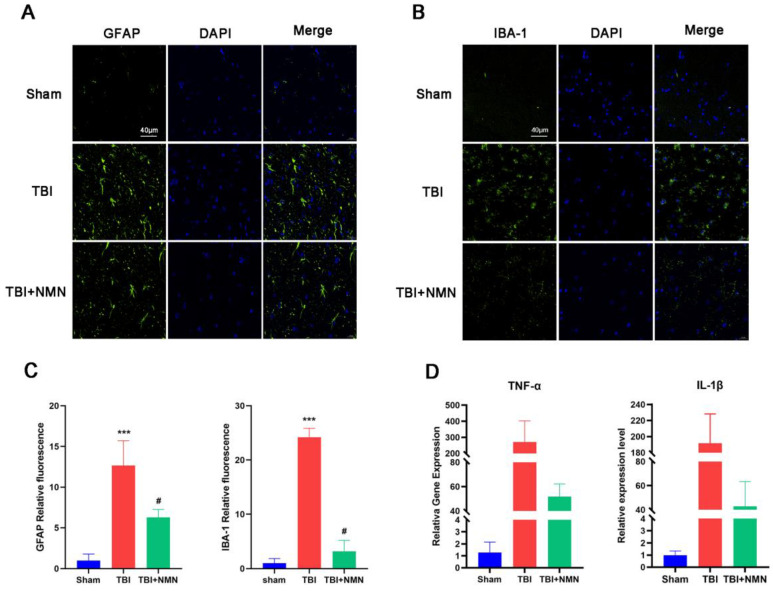
** NMN treatment alleviated microglial and astrocyte activation and inhibited inflammatory response in hippocampal CA1 at 8 days after TBI. A)** Representative fluorescence images for staining of GFAP in CA1 region. Scale bar = 40 μm (n =3 per group). **B)** Representative fluorescence images for staining of IBA-1 in CA1 region. Scale bar = 40 μm (n =3 per group). **C)** quantitative analysis of GFAB and IBA-1 fluorescence intensity in the DG. **D)** RT-qPCR of IL-1β and TNF-α mRNA expression levels at 24h after TBI (n =3 per group). (*p < 0.05, **p < 0.01, and ***p < 0.001 TBI vs Sham group; #p < 0.05 and ##p < 0.01 TBI vs TBI +NMN group).

In the behavioral experiment completed, NMN treatment showed a neuroprotective effect on TBI. Previous studies showed the neuroinflammation, which is closely related to the activation of microglia and astrocyte activation, is one of the important mechanisms of TBI secondary injury 33-35. Therefore, we further administrated whether NMN affected the activation of glial cells after TBI. The activated astrocytes maker GFAP and the microglia maker Iba-1 immunostaining showed that NMN treatment significantly reduced the TBI-induced activated astrocytes and microglia in the CA1 area of hippocampus, and the quantitative analysis of fluorescence intensity showed the same result (*P* < 0.05) (**Fig. 3A-C**).

TNF-α and IL-1β as representative inflammatory factor in TBI, their expression level reflects the degree of inflammatory response. We detected the expressions of TNF-α and IL-1β by RT-qPCR. The finding illustrated that the expression of TNF-α and IL-1β genes in hippocampus of TBI+NMN group was significantly lower than TBI group (*P* < 0.05) (**Fig. 3D**). These results indicated NMN can relieve the inflammatory response after TBI.

### NMN decreased neuronal apoptosis after TBI

**Figure 4:**
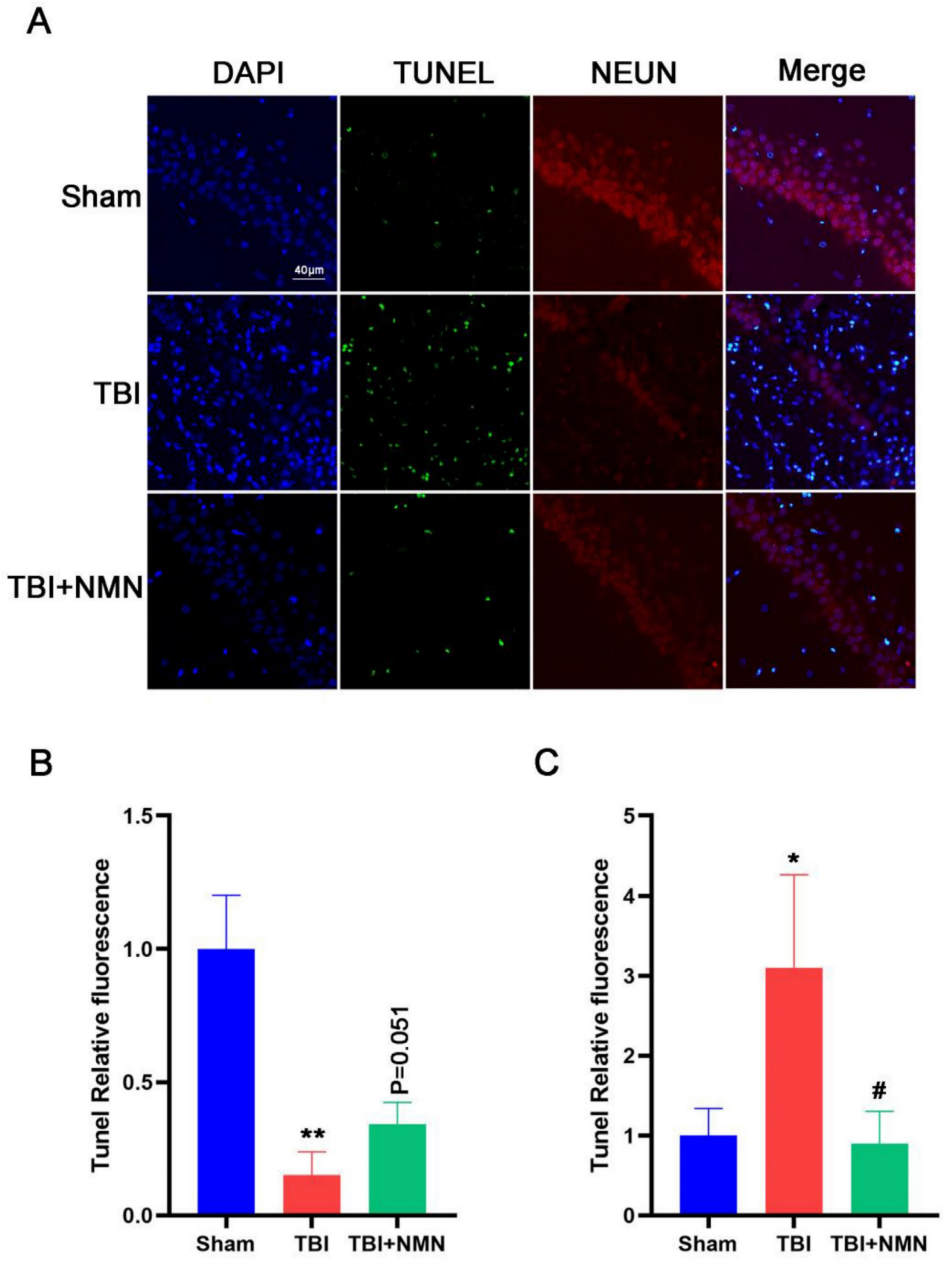
** NMN decreased neuronal apoptosis at 8 days after TBI. A)** Representative images of NEUN, TUNEL, and DAPI costaining of brain sections. Scale bar: 40 nm (n =3 per group). **B)** Quantitative analysis of NEUN fluorescence intensity in CA1 region (n =3 per group). **C)** Quantitative analysis of TUNEL fluorescence intensity in CA1 region. (n =3 per group, *p < 0.05, **p < 0.01, and ***p < 0.001 TBI vs Sham group; #p < 0.05 and ##p < 0.01 TBI vs TBI +NMN group).

The improvement of neurological functions after TBI is not only related to the reduction of inflammation, but also related to the damage of neurons directly. Therefore, we next assessed neuronal apoptosis by TUNEL assay. Brain samples were taken for double immunofluorescence staining to detect the co-expression of TUNEL and NEUN in CA1 area (**Fig. 4A**). Consistent with the neuroinflammation result, the NEUN fluorescence intensity in hippocampus were decreased (*P* < 0.05) and the TUNEL fluorescence intensity were increased after TBI compared with the sham group (P < 0.05). And NMN administration significantly decreased the fluorescence intensity of TUNEL after TBI (*P* < 0.05). The fluorescence intensity of NEUN in TBI+NMN group also increased significantly compared with the TBI group (**Fig. 4B, C**). These results demonstrated that NMN treatment was able to reduce neuronal apoptosis.

### Hippocampal transcriptome analysis of three groups

**Figure 5:**
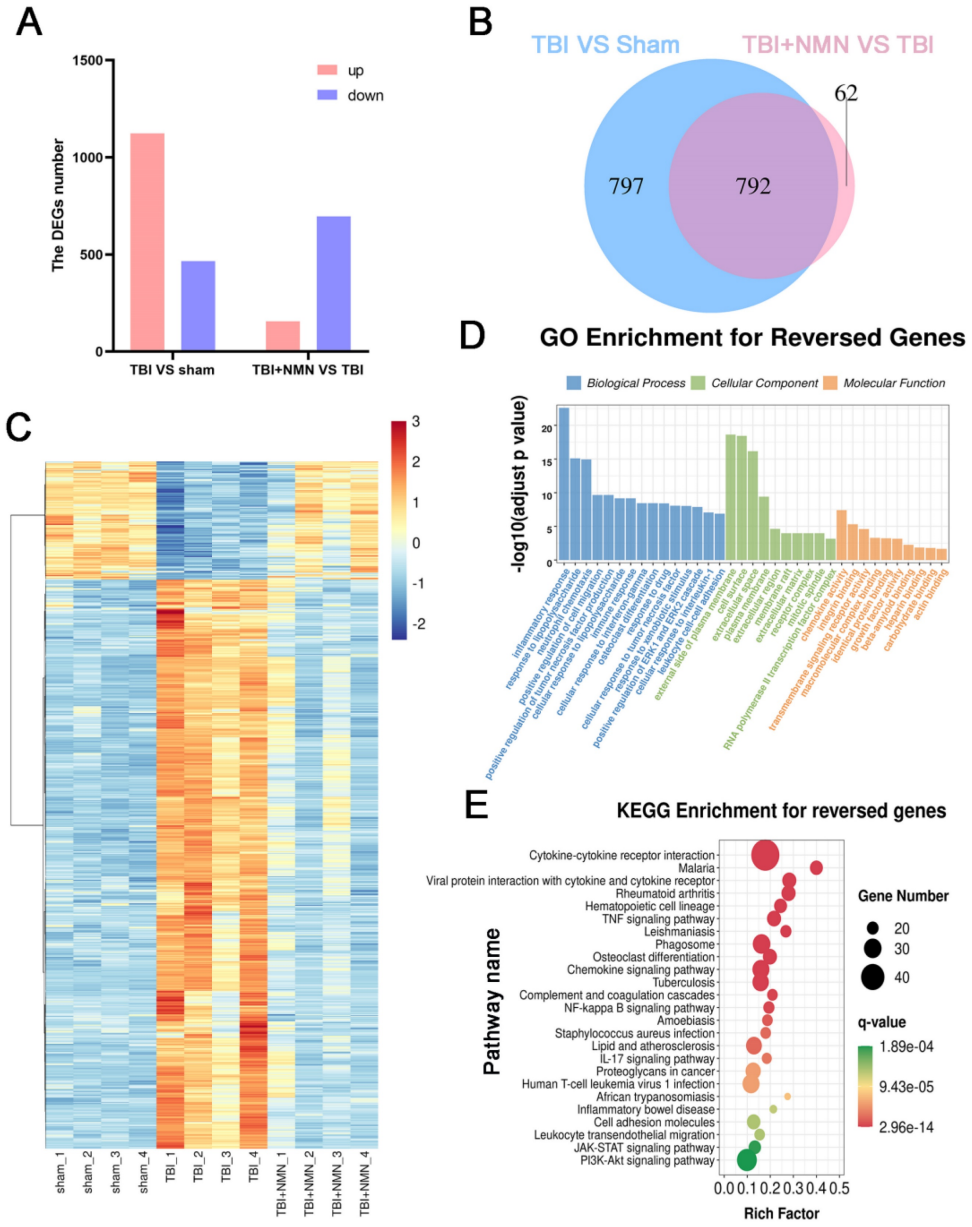
** RNA-seq analysis on Sham, TBI, and TBI +NMN in rat 24h post-TBI (n=4 per group). A)** Histogram of deferentially expressed genes (DEGs) between different groups. **B)** Venny analysis between DEGs in TBI vs Sham and DEGs in TBI+NMN vs TBI. **C)** Expression heatmap of 792 reversed genes. **D and E)** GO enrichment analysis and KEGG enrichment analysis based on the reversed genes.

To further probe the related mechanism of neuroprotective effect of NMN after TBI, transcriptome sequencing was performed on hippocampus of Sham, TBI, and TBI+NMN groups. The sequencing results showed that there were 1589 differentially expressed genes (DEGs) in TBI vs sham, including 1123 up-regulated and 466 down-regulated, and 157 up-regulated and 697 down-regulated were accessed due to the effect of NMN treatment (|log2FC| > 1 and p < 0.01) (**Fig. 5A and Table S1**). Of the 1589 DEGs genes that appeared after TBI, 792 were reversed in the TBI+NMN group, including 656 up-regulated and 136 down-regulated ones (**Fig. 5B and Table S1**). Hierarchical cluster analysis of 792 reversed genes shows that gene expression has been significantly reversed after TBI and treatment (**Fig. 5D**).

Gene ontology (GO) and KEGG enrichment analysis of these common differential genes were carried out to explore the function of them. The 792 reversed DEGs were enriched in inflammation related GO items, such as inflammatory response, positive regulation of cell migration, positive regulation of tumor necrosis factor production, immune response, cellular response to tumor necrosis factor, positive regulation of ERK1 and ERK2 cascade and cellular response to interleukin-1 **(Fig. 5C and Table S2)**. They were also enriched in Cytokine-cytokine receptor interaction, TNF signaling pathway, Phagosome, Chemokine signaling pathway, NF-kappa B signaling pathway, Cell adhesion molecules, JAK-STAT signaling pathway, PI3K-Akt signaling pathway and some immunity-related pathway** (Fig. 5E and Table S2)**. The above results suggested that NMN may exert neuroprotective effects via influencing inflammation and immunity related signaling pathways.

**Figure 6:**
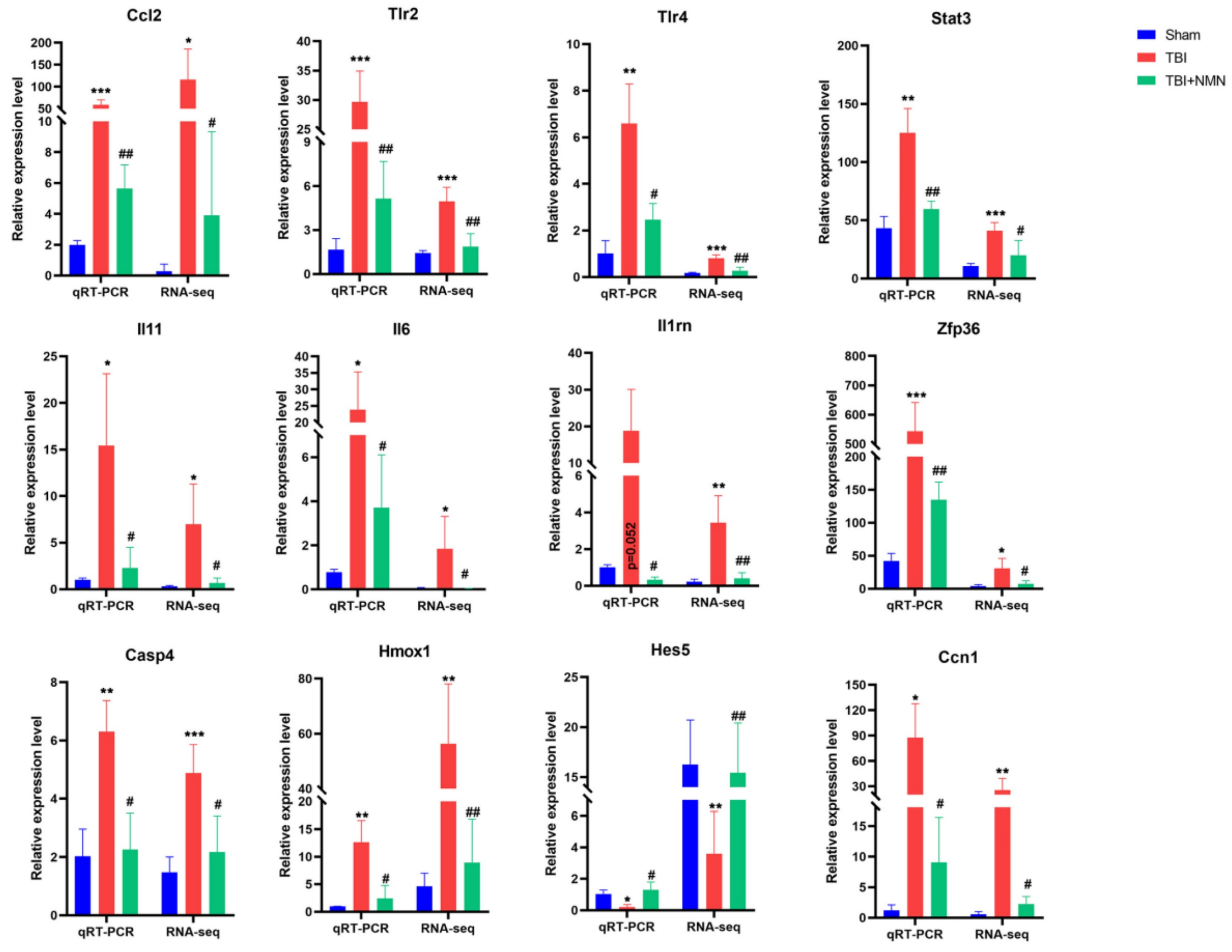
RT-qPCR of Ccl2, Tlr2, Tlr4, Stat3, Il11, Il6, Il1rn, Zfp36, Casp4, Homx1, Hes5 and Ccn1 mRNA expression levels at 24h after TBI. (n = 6 per group, *p < 0.05, **p < 0.01, and ***p < 0.001 TBI vs Sham group; #p < 0.05 and ##p < 0.01 TBI vs TBI +NMN group).

At last, in order to confirm the reliability of RNA sequencing results, a series of genes were selected to detect their expression levels using RT-qPCR. As showed in **Fig. 6**, 10 genes (*Ccl2*, *Trl2, Trl4, Stat3, Il11, Il6, Il1rn, Zfp36, Casp4, Hmox1, and Ccn1*) were up-regulated in TBI group compared to sham group, and were significantly down-regulated in TBI+NMN group compared to TBI group. Moreover, *Hes5* was down-regulated in TBI group compared to sham group and reversed by NMN treatment. The sequencing results and PCR results were mutually verified.

## Discussion

TBI is initially caused by mechanical damage, but its secondary damage is complex and diverse. Substantial evidence suggested that cellular cascade of inflammation participates in secondary brain injury 36-39. However, short-term inflammation after TBI is protective, while long-term, intense inflammation can damage the brain 40. And a number of studies have reported that NAD^+^ play critical roles in many biological processes including metabolism, inflammation, and stress and damage response resulting from its consuming enzymes 41, 42. Our study demonstrated that the administration of NMN, a NAD^+^ intermediates, is capable of alleviating TBI-associated neurological impairment by mitigating neuronal inflammation. In the present study, we first examined the effect of NMN treatment on the pathological changes after TBI. We found that NMN reduced the death of nerve cells, maintained the structure of hippocampus, and alleviated the brain edema after TBI, which proved that NMN can effectively reduce brain damage caused by TBI. Previous studies have shown that the degree of neuronal death in the hippocampus due to contusion, inflammation, edema and other reasons affects the postoperative recovery of TBI 43, 44. Thus, we further investigated the neurological function of NMN in TBI rats. We found that NMN-treated TBI rats substantially got lower mNSS scores and lower escape latency compared to TBI rats. Zhao's study reported that NAD+ ameliorate cognitive impairment in chronic cerebral hypoperfusion (CCH) models 45, which is consistent with our results. These findings proved that NMN can play a protective role in brain neurons after TBI.

Astrocytes and microglia which play crucial roles in the inflammatory response of the central nervous system can lead to neurodegenerative diseases 46. According to our findings, decreased GFAP and Iba-1were observed under NMN administration. Pro-inflammatory response astrocytes induce proinflammatory factors (e.g., IL-1β, TNF-α) that are known to have deleterious functions 47. Besides, microglia were reported have ability to reduce clearance effect, which leads to the aggravation of nerve cell damage 48. Moreover, NAD+ treatment have been reported to reduce CCH-induced microglia and subsequent production of pro-inflammatory factors 45. Similarly, TBI rats in our study exhibited activated TNF-α and IL-1β that were mitigated by NMN treatment. Additionally, Post-traumatic neuroinflammation have been reported to significantly contribute to the neuronal death observed after neurotrauma 49. TUNEL assay in our study also demonstrated that NMN treatment decreased TBI-induced neuronal apoptosis. Given these results, we hypothesized that the improvement of neurological function in TBI rats may be result from the fact that NMN alleviated neuroinflammation.

To further investigated the related mechanism of NMN in TBI, we identified differentially expressed genes in TBI by RNA sequencing and explored genes and enrichment pathways for NMN reversal. We found 1589 genes were changed in TBI and 792 genes were reversed by NMN administration. And GO analysis showed that the different reversed genes were strongly related to inflammatory response, which was consistent to our experiment findings. Our data proved that MNM treatment reversed the elevated CCL2 which induced by TBI. Zhao et al. reported CCL2 which was derived from microglia, astrocytes and neurons can aggravate tissue damage by conducting secondary inflammatory response 50. In addition, the reversal of proinflammatory cytokines such as IL-6, IL-11 and IL1rn also proved that NMN could reduce the level of inflammation after TBI. Moreover, we also found that Toll-like receptors TLR2 and TLR4 were also reduced by NMN treatment. Tu et al. found that inhibition of TLR2/4-NF-κB signaling reduced brain damage and protected nerves 51, 52. Lin et al. also revealed that TLR2/4-NF-κB signaling has an inhibitory effect in the later stage of TBI 53. Wan et al. reported NMN prevented DIC in rats by inhibiting NLRP3 inflammatory body activation 54. These findings were in agreement with our results. Similarly, KEGG analysis also showed that the NF-kappa B signaling pathway, Jak-STAT signaling pathway, TNF signaling pathway were significantly enriched. Besides, present studies have revealed that oxidative and inflammation are indispensable in TBI 55, 56. The increased NAD+ can preserve cellular respiration. reduce oxidative stress, and further alleviate inflammation 57.

## Conclusion

In summary, this study provide evidence that NMN administration alleviated TBI-associated neurological impairment by anti-neuroinflammation bioactivities. Besides, the underlying molecular mechanisms of these beneficial effects may involve TLR2/4-NF-κB signaling. Our data might provide a novel therapeutic strategy for TBI. However, the precise mechanism on NMN still needs to be explored in further study.

## Supplementary Material

Supplementary table 1.

Supplementary table 2.

## Figures and Tables

**Table 1:** Primer sequences used in this study

Gene	Species	Primer sequence
Ccl2	RAT	Forward: 5' -CCACTCACCTGCTGCTACTCATTC -3'
		Reverse: 5' -CTGCTGCTGGTGATCCTCTTGTAG -3'
Trl2	RAT	Forward: 5' - GGAATCAACACAATAGAGGGAG -3'
		Reverse: 5' -CTGAACCAGGAGGAAGATAAAC -3'
Trl4	RAT	Forward: 5' - CCTCCCTGGTGTTGGATTTAC -3'
		Reverse: 5' -AGATGCTTTCTCCTCTGCTGTA -3'
Stat3	RAT	Forward: 5' - CAATACCATTGACCTGCCGAT -3'
		Reverse: 5' - GAGCGACTCAAACTGCCCT -3'
Il11	RAT	Forward: 5' - TGGGGACATGAACTGTGTTTGT -3'
		Reverse: 5' -TGCAAAGATCCCAGTGTCCC -3'
Il6	RAT	Forward: 5' - ACTTCCATCCAGTTGCCTTCTTGG -3'
		Reverse: 5' - TTAAGCCTCCGACTTGTGAAGTGG -3'
Il1rn	RAT	Forward: 5' - TCCTTCTCATCCTTCTGTTTCGT -3'
		Reverse: 5' - AGTGATGTTAACCTCCTCCAGC -3'
Zfp36	RAT	Forward: 5' - AGCGGCTCCCAGATCAACT -3'
		Reverse: 5' - CGAAAGCGAAGGCGTTGTTA -3'
Casp4	RAT	Forward: 5' - ACAAACACCCTGACAAACCAC-3'
		Reverse: 5' - CACTGCGTTCAGCATTGTTAAA -3'
Hmox1	RAT	Forward: 5' - CTAAGACCGCCTTCCTGCTC -3'
		Reverse: 5' -TGCAGAGGTAGTATCTTGAACC -3'
Hes5	RAT	Forward: 5' - CCAAGGAGAAAAATCGACTGCG -3'
		Reverse: 5' -CGAAGGCTTTGCTGTGCTTC -3'
Ccn1	RAT	Forward: 5' - GGCGTTGACAGTACGTTTGG-3'
		Reverse: 5' -AGAGGCTTCCTGTCTTTGGC -3'
TNFα	RAT	Forward: 5' - CTCCAGGCGGTGCCTATG -3'
		Reverse: 5' -GGGCCATAGAACTGATGAGAGG -3'
IL1β	RAT	Forward: 5' - GCACACCCACCCTGCA -3'
		Reverse: 5' -ACCGCTTTTCCATCTTCTTCTT -3'
